# Beetroot-Pigment-Derived Colorimetric Sensor for Detection of Calcium Dipicolinate in Bacterial Spores

**DOI:** 10.1371/journal.pone.0073701

**Published:** 2013-09-03

**Authors:** Letícia Christina Pires Gonçalves, Sandra Maria Da Silva, Paul C. DeRose, Rômulo Augusto Ando, Erick Leite Bastos

**Affiliations:** 1 Centro de Ciências Naturais e Humanas, Universidade Federal do ABC, Santo André, SP, Brazil; 2 Biosystems and Biomaterials Division, Chemical Science Technology Laboratory, National Institute of Standards and Technology. Gaithersburg, Maryland, United States of America; 3 Departamento de Química Fundamental, Instituto de Química, Universidade de São Paulo, São Paulo, SP, Brazil; Loyola University Medical Center, United States of America

## Abstract

In this proof-of-concept study, we describe the use of the main red beet pigment betanin for the quantification of calcium dipicolinate in bacterial spores, including *Bacillus anthracis*. In the presence of europium(III) ions, betanin is converted to a water-soluble, non-luminescent orange 1∶1 complex with a stability constant of 1.4×10^5^ L mol^–1^. The addition of calcium dipicolinate, largely found in bacterial spores, changes the color of the aqueous solution of [Eu(Bn)^+^] from orange to magenta. The limit of detection (LOD) of calcium dipicolinate is around 2.0×10^–6^ mol L^–1^ and the LOD determined for both spores, *B. cereus* and *B. anthracis*, is (1.1±0.3)**×**10^6^ spores mL^–1^. This simple, green, fast and low cost colorimetric assay was selective for calcium dipicolinate when compared to several analogous compounds. The importance of this work relies on the potential use of betalains, raw natural pigments, as colorimetric sensors for biological applications.

## Introduction

Bacteria of genus *Bacillus* can assume a dormant and resistant spore form (i.e., *endospore*) in order to survive harsh environmental conditions. Although some bacterial spores contribute to human activities, e.g., *B. thurigiensis* is used as a pesticide, other species, such as *B. anthracis*, *B. cereus*, pose a serious risk to human health [1]. *Bacillus anthracis* has emerged as a bioterrorism agent because of its high stability and virulence [2], [3] and became a public safety concern again after the anthrax attack in 2001 [4]. The infection usually takes place through skin contact with infected animals or animal products, but can also occur by inhalation or ingestion of spores [5]. Another example, *Bacillus cereus,* has negative economic impacts because this pathogenic bacterium can grow on food [6]. Consequently, there is a need for early detection of *Bacillus* species to ensure human health and safety.

Calcium dipicolinate (CaDPA) is the major component (up to 15% dry weight) of the bacterial spore core [7]. It stabilizes the bacterial DNA, contributes to the overall chemical and heat resistance of the spore and is released during germination [8], [9]. Endospores also release CaDPA upon thermal treatment or in the presence of reactivation agents that induce bacterial germination, such as inosine and L-alanine [10]. Although there are several methods for the detection of *B. anthracis* [11], [12], the simple and fast detection of dipicolinic acid (DPA) using luminescent lanthanide has been promising [9], [13], [14], [15].

Betanin (**Bn**) is the non-toxic, water-soluble pigment responsible for the deep red–magenta color of the red beet (λ = 536 nm, *h*° = 336, ε = 6.5×10^4^ L mol^–1^ cm^–1^) [16], [17]. **Bn** is a food colorant (additive E-162) with a high antioxidant capacity [18], [19], [20], which has been used in several different applications such as dyes in solar cells [21], [22] or as a starting material in the semi-synthesis of fluorescent probes for the live-imaging of *Plasmodium*-infected red blood cells [23]. In this proof-of-concept study, betanin is used as a ligand in a new Eu^III^ complex, which is sensitive to CaDPA but not to analogous compounds. In order to demonstrate the applicability of the method in detecting CaDPA, we used two representative Bacillus species (*B. anthracis* and *B. cereus* spores) as test samples.

## Material and Methods

### Complexation Studies

#### Determination of equilibrium constants

The stoichiometry of the Eu^III^/**Bn** complex was determined using the molar-ratio method (Yoe and Jones’ method) [24]. The concentration of a solution of **Bn** in MOPS buffer pH = 7.5 (10 mmol L^–1^) was kept constant (5.57 µmol L^–1^) and a variable amount of Eu^III^ (0.3 to 22 equiv) was added. Experiments were carried out independently at 25±1°C in quartz cuvettes (o.p. 10 mm) with a final volume of 2 mL. The mole ratio of the metal ion to **Bn** was plotted versus absorbance at 536 nm and tangents were drawn. The perpendicular line located at the intersection of the tangents was drawn to the mole ratio axis showing the Eu^III^/**Bn** ratio.

Stability constants were determined using a simple metal–ligand complexation model [25] considering a 1∶1 stoichiometry (Eq. (1)):
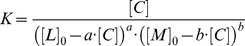
(1)where, L: ligand; M: metal; C: complex; a and b are the stoichiometric factors; [L]_0_ and [M]_0_: initial total concentration of the ligand and the metal, respectively; [L], [M] and [C]: equilibrium concentration of the ligand, the metal and the complex, respectively.

For the determination of *K* by UV/Vis spectrometry, it is necessary to determine the [C]. In case the metal does not absorb at the wavelength λ, [C] can be determined using Eq. 2. The mathematical derivation of this method and additional experimental details are presented in the File S1.
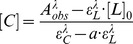
(2)where, A^λ^_obs_ is the observed absorbance at a given wavelength, ε^λ^_L_ and ε^λ^_C_ are the molar absorption coefficient of the ligand and complex at the same wavelength, respectively.

The data obtained with the titration experiment were used for the determination of the lanthanide–betalain stoichiometry as well as for the determination of [C] using Eq. (2). The stability constant was determined by averaging the values obtained using Eq. (1) for each concentration of lanthanide (Table S1).

#### Calibration curve and limit of detection

In a 96 well microplate, solutions of [Eu(Bn)]^+^ were prepared by adding different amounts of a 1 mmol L^–1^ solution of EuCl_3_ in MOPS buffer to a solution of **Bn** in water. Next, a defined volume of a solution of CaDPA (1 mmol L^–1^) was added and the volume in each well was adjusted to 200 µL using MOPS buffer pH = 7.5. Final concentrations are as follows: [**Bn**] = 5.6 µmol L^–1^, [Eu^III^] = 5.6 to 33.4 µmol L^–1^, [CaDPA] varied from 0.6 up to 120 µmol L^–1^ depending on the [Eu^III^]. The absorbance at 536 nm was registered either in the absence or in the presence of variable amounts of CaDPA and used to calculate the variation in the response (ΔAbs^536 nm^ = ΔAbs^536 nm^_CaDPA_ – ΔAbs^536 nm^_Control_). The calibration curve was constructed by plotting the concentration of CaDPA in mol L^–1^ (*y*) versus the ΔAbs^536 nm^ (*x*). The data points in the linear interval of the sigmoidal curve were submitted to linear regression analysis.

The limit of detection (LOD) was determined using a procedure based on a linear model in different zones of the sigmoid response function; namely, the maximum and minimum slope (at low analyte activity) of the response function is fitted to a linear function by a statistical lack-of-fit test. Thus, the LOD is defined as the activity of the analyte that corresponds to the intersection between the two straight lines at the higher and lower slope (signal-to-noise ratio of 3) [26].

### Microbiological Methods

#### Bacillus cereus sample preparation

Spore suspensions of *Bacillus cereus* ATCC 10987 were prepared by growing a uniform lawn of spores on solid PGSM [27]. After 7 d of growth (3 d at 37°C and the remainder at 21°C), plates were examined daily for spore formation by removing a small amount of spores and examining with phase microscopy (Olympus AX-70, Olympus America Inc, Center Valley, PA). Once 95% spore formation was noted, spores were suspended from the agar surface by pouring 2.0 mL of sterile water and gently scraping the agar surface with a clean, sterile glass cell spreader. Spore preparations were then washed 5 times in sterile water and then stored in water suspension at 4°C.

#### Suspensions of Green Fluorescent Protein (GFP)-labeled bacillus anthracis

*Bacillus anthracis* Sterne pAFp8gfp were prepared by growing a uniform lawn of spores on a modified Schaeffer media [28]. After 5 d of growth at 32°C, plates were examined daily for spore formation by removing a small colony of growth and examining with phase microscopy. Once 95% spore formation was noted, spores were harvested from the agar surface by pouring 2.0 mL of sterile water and gently scraping the agar surface with a clean, sterile glass cell spreader. Spore suspensions were washed 7 times with water by centrifugation (1,500×g, 2 min) and stored at 4°C as a water suspension.

#### Spore cleaning

Water suspensions of *B. cereus* or *B. anthracis* (200 µL) were washed 3 times with water by centrifugation (10,000×g, 2 min) and suspended in 40% v/v ethanol to eliminate any potential presence of CaDPA from germinated spores in the stock suspension. In addition, the samples were heated for 25 min at 65°C to inactivate any vegetative cells or germinated spores in the sample. The sample was named stock suspension and used to prepare a 12-fold diluted working suspension in water.

#### Determination of spore concentration

*B. cereus* and *B. anthracis* working suspensions were quantified by serial dilution in phosphate buffered saline pH = 7.4 containing 0.4% v/v Tween 80 (PBST), plated onto LB agar using the drop plate method (5 drops of 10 µL per dilution) and cultured for about 16 h at 30°C. Colony forming units (CFU) were enumerated and used to calculate the spore concentration in the working suspension [27].

#### Release of CaDPA from spores

Working suspensions (100 µL) of *B. cereus* ((1.3±0.2)×10^9^ spores mL^–1^) or *B. anthracis* ((1.4±0.3)×10^8^ spores mL^–1^ or (3.5±0.8)×10^8^)) were autoclaved (20 min, 121°C) followed by sonication for 30 min. The samples were centrifuged (10,000×g, 2 min) and the supernatant containing CaDPA was transferred to another microfuge tube and used in the measurements.

### Curve Fitting and Statistical Analysis

All values are expressed as mean ± standard deviation (*sd*) of three completely independent replicates, except when indicated. Statistical data analysis was performed by one-way analysis of variance (ANOVA) and the level of statistical significance was taken to be *p*<0.05. Curve fitting and statistical calculations were carried out using the software Origin (version 8.5; OriginLab: Northampton, MA, USA, 2011).

Linear regression analysis was carried out by the method of least squares. Sigmoidal curves, with a lower boundary near the background response and an upper asymptote near the maximum response, were fitter using the 4-parameter logistic model (Eq. (3)) [29].
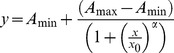
(3)


Where, *y* is the response, *A_max_* is the response at infinite analyte concentration, *A_min_* is the response at zero analyte concentration, *x* is the analyte concentration, *x_0_* is the inflection point on the curve (IC_50_), and *α* is a slope factor.

## Results and Discussion

### Complexation of Bn and Eu^III^

**Figure 1:**
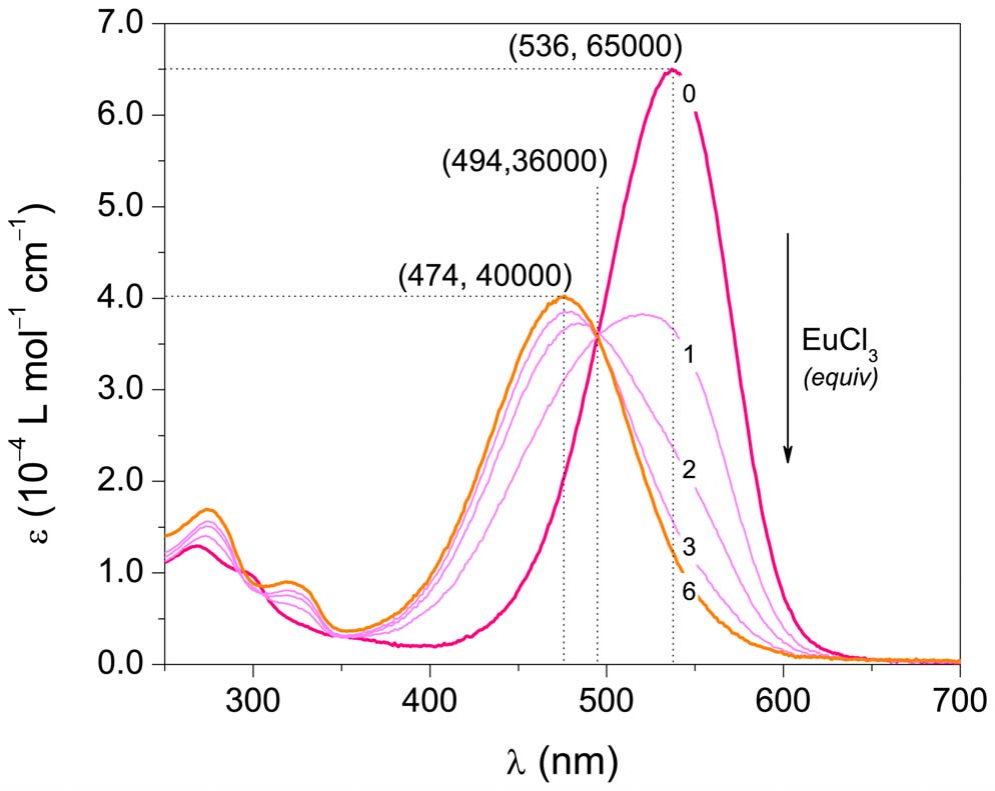
Absorptivity profile for the formation of [Eu(Bn)]^+^ by the addition of EuCl_3_ to a solution of Bn in MOPS buffer pH = 7.5. The numbers in the curves indicate the concentration of EuCl_3_ in equivalents of Bn.

Most transition metals have been found to catalyze the decomposition of betanin (**Bn**) in aqueous solution. However, **Bn** has been described to form complexes with transition metals such as Cu^I^, Cu^II^ and Hg^II^ in near-neutral aqueous media [30]. Interestingly, complexation of **Bn** and Eu^III^ occurs spontaneously in MOPS buffer pH = 7.5, resulting in the bright orange and relatively stable [Eu(Bn)]^+^ complex (λ = 474 nm, *h*° = 53, ε^474^ ^nm^ = 4.0×10^4^ L mol^–1^ cm^–1^, *k*_obs_ = 2.7 10^–4^ min^–1^ in MOPS buffer pH = 7.5 at 25°C, Figure 1 and Figure S1).The Eu^III^–betanin stoichiometry was determined to be 1∶1 by using the molar-ratio method (Figure S2). Contrary to our expectations, the complex is not fluorescent in water probably due to water coordination to Eu^III^, which favors the non-radiative deactivation of the excited lanthanide [31]. The stability constant of the [Eu(Bn)]^+^ complex was determined to be (1.4±0.6)×10^5^ L mol^–1^ (Table S1) using Eq. (1) and is similar to that reported for the complexation of Ca^II^ and DPA (log *K* = 5.4, *K* = 2.51×10^5^ L mol^–1^) [32].

Despite our efforts, we were unable to obtain a single crystal of the [Eu(Bn)]^+^ complex suitable for crystallographic structure determination. However, insights into the structure of the complex were obtained by Raman spectroscopy associated with theoretical calculations. Figure S3 depicts the resonance Raman spectra of **Bn** and [Eu(Bn)]^+^ excited at 514.5 nm and 476.5 nm, respectively, as well as the theoretical Raman spectra of **Bn** (computational details and Cartesian coordinates are in File S1). The four intense Raman signals at 1610, 1518, 1506 and 1394 cm^–1^ in the spectrum of **Bn** can be assigned to ν(C = N)+ν(C = C)+φ_8a_, φ_19a_+ν(C = N), ν(C = C)_pyr_+ν(C = N), and ν(C = N), respectively. The observed resonance Raman enhancement profile is in accordance to HOMO-LUMO DFT calculations for the dianionic form of **Bn** (p*K*_a1_ = 3.4; p*K*_a2_ = 8.5, [33]) that show a charge transfer from the aromatic ring to the dihydropyridyl moiety [21], [34]. Raman signals of [Eu(Bn)]^+^ at 1616, 1529, and 1436 cm^–1^ are shifted to higher wavenumbers compared to that of **Bn** probably because the electronic delocalization of the π-system decreases upon complexation, i.e., bond orders increase. Furthermore, the Raman band of **Bn** at 1155 cm^–1^ (vibrational mode involving the tetrahydropyridyl N–H bending, δ(NH)) is missing in the Raman spectra of the [Eu(Bn)]^+^ complex. These results are in agreement with the blue shift (60 nm, 2440 cm^–1^) in the maximum absorption wavelength upon the [Eu(Bn)]^+^ complex formation and allow us to infer that Eu^III^ complexation is likely to occur through the 2,6-dicarboxyl-1,2,3,4-tetrahydropyridine moiety.

### Determination of CaDPA

**Figure 2:**
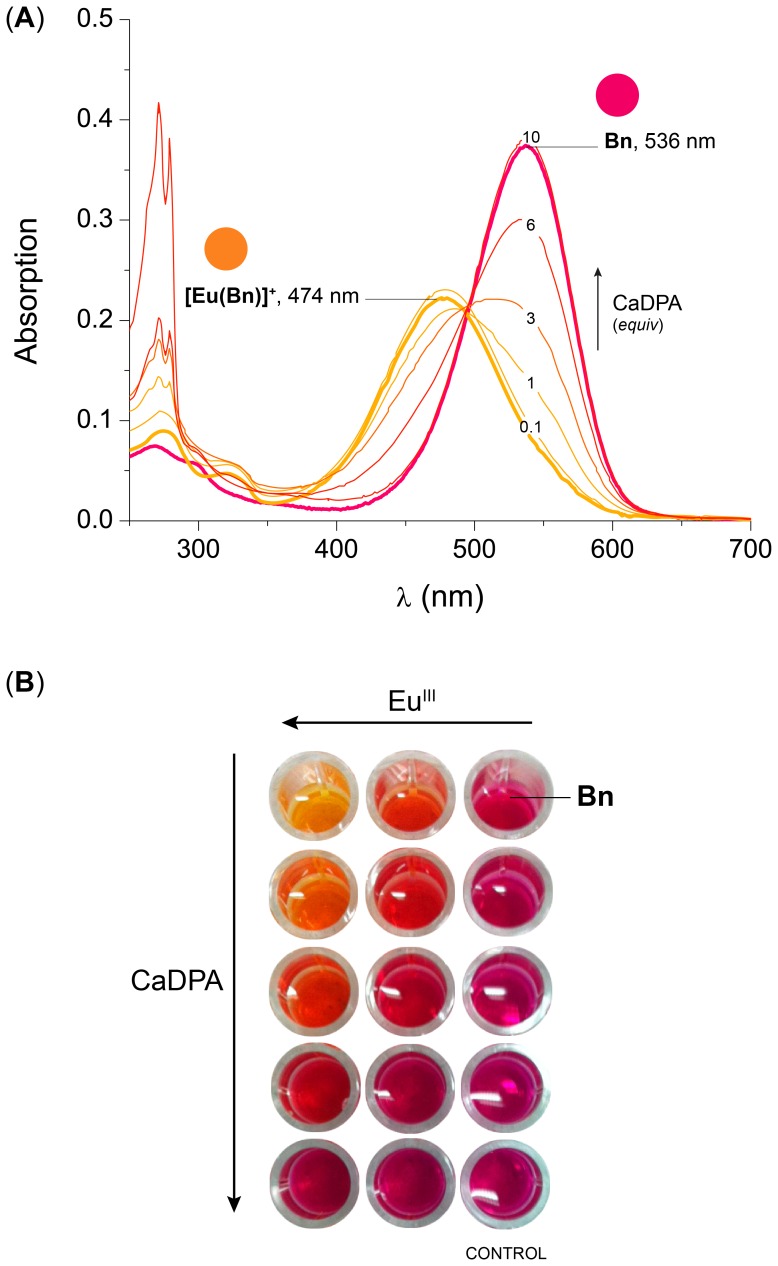
Effect of the addition of CaDPA on an aqueous solution of [Eu(Bn)]^+^. (A) Absorption profile for the formation of **Bn** by the addition of CaDPA to a solution of [Eu(Bn)]^+^; The numbers in the curves indicate the concentration of CaDPA in equivalents of **Bn**. (B) Picture of a microplate containing **Bn** and increasing amounts of EuCl_3_ and CaDPA, the background was removed for clarity. [**Bn**] = 5.8 µmol L^–1^, [EuCl_3_] = 34.8 µmol L^–1^ (6 equiv) in MOPS buffer pH = 7.5.

CaDPA competes with **Bn** for Eu^III^ ions. The stability constants reported for complexes of type [Eu(DPA)*_n_*]^3−2*n*^ (K_1_ = 6.92×10^8^ (*n* = 1); K_2_ = 1.38×10^7^ (*n* = 2); K_3_ = 3.23×10^5^ (*n* = 3) [35] are higher than the stability constant determined for [Eu(Bn)]^+^ (i.e., *K*_1_ = 1.4×10^5^). Therefore, the reaction of CaDPA with the [Eu(Bn)]^+^ complex releases **Bn** due to the formation of [Eu(DPA)*_n_*]^3−2*n*^ complexes, changing the color of the solution from orange to red-magenta (Figure 2).

**Figure 3:**
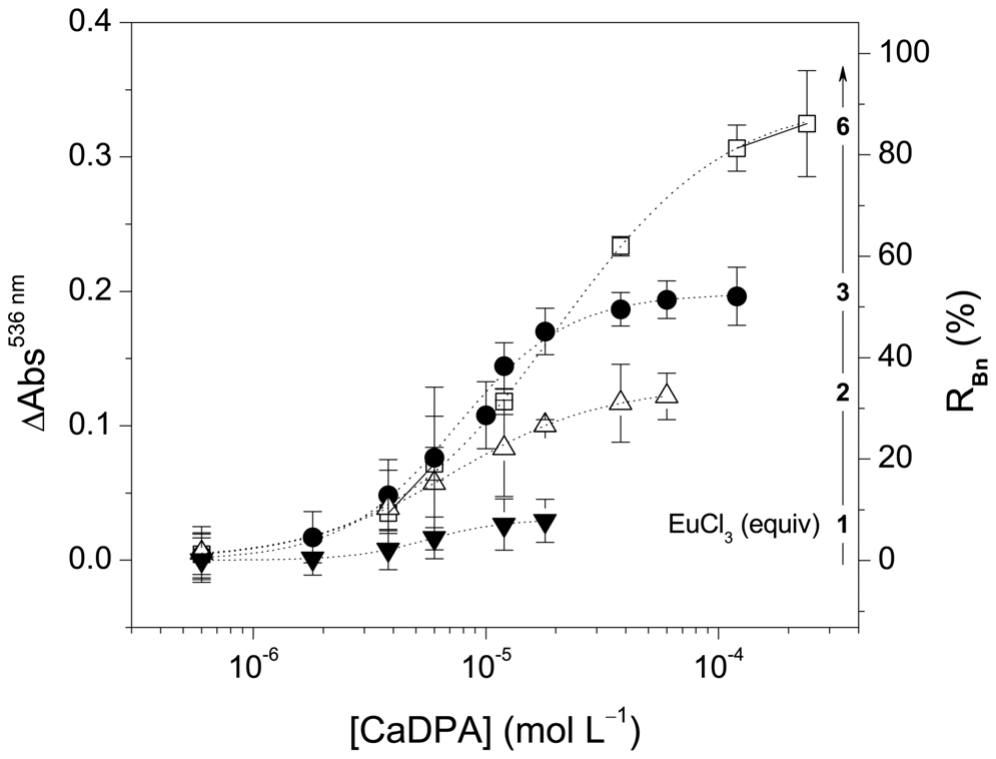
Effect of the concentration of Eu^III^ on the quantification of CaDPA. The variation of the absorbance at 536 nm and the percent recovery of **Bn** are plotted against the concentration of CaDPA (log scale). Lines indicate the fitting of data using Eq. (3) with parameter *A_min_* set to zero; [**Bn**] = 5.8 µmol L^–1^ in MOPS buffer. LOD are: 6 equiv, (2.8±1.5)×10^–6^ mol L^–1^; 3 equiv, (2.2±1.1)×10^–6^ mol L^–1^; 2 equiv, (1.4±0.9)×10^–6^ mol L^–1^ and 1 equiv, (1.7±0.8)×10^–6^ mol L^–1^.

The effect of the amount of Eu^III^ (1, 2, 3 or 6 equiv) on the quantification of CaDPA was investigated. All curves depicted in Figure 3 show the sigmoidal profile characteristic of ligand binding assays [29]. The limit of detection (LOD) in this experimental condition is around 2×10^–6^ mol L^–1^, independent of the concentration of Eu^III^. However, the recovery of **Bn** (R**_Bn_**), i.e., the ratio between the initial absorbance at 536 nm before addition of Eu^III^ and CaDPA and that after the addition of mole excess of CaDPA to the [Eu(Bn)]^+^ complex, increases with the amount of Eu^III^ (Figure 3). This is probably related to the decrease in the concentration of unbound **Bn** in equilibrium caused by the increase in the concentration of Eu^III^. Furthermore, the variation in absorbance increases with the increase of the concentration of Eu^III^; consequently, the signal-to-noise ratio is improved using large mole excess of the lanthanide.

**Figure 4:**
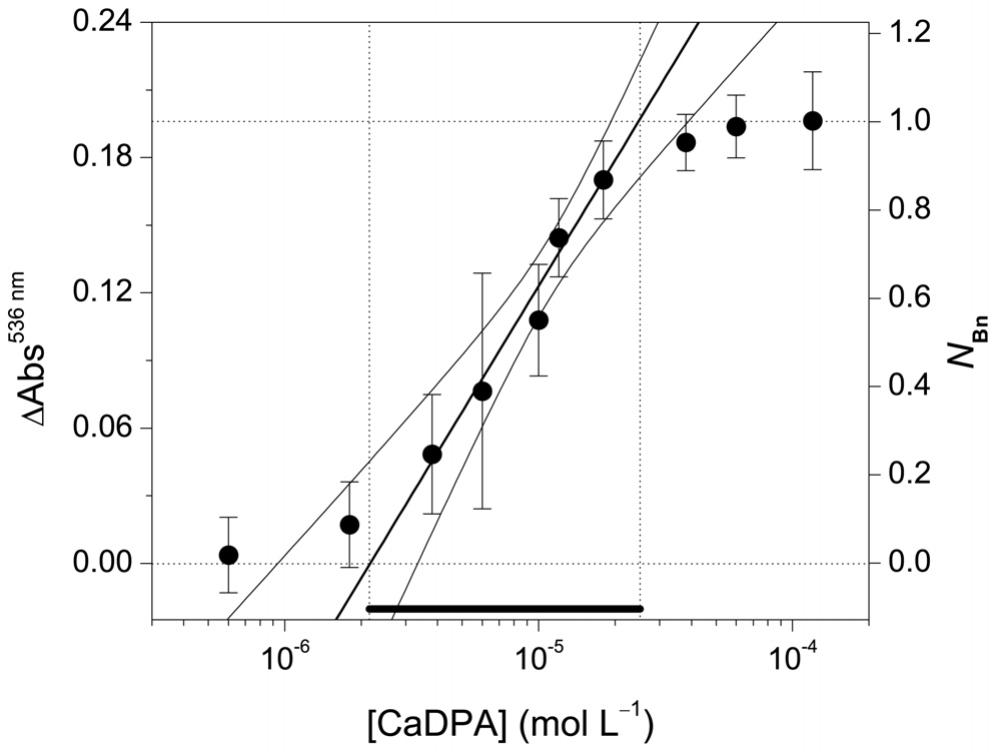
CaDPA concentration (log scale) plotted against the variation in the absorbance at 536 nm (ΔAbs^536 nm^) and the normalized response (*N*_Bn_). The diagonal straight-line shows when the plotted parameters are linearly correlated. Curved lines are the confidence bands at the 95% level. Error bars represent the *sd* of triplicates. [**Bn**] = 5.8 µmol L^–1^, [EuCl_3_] = 17.4 µmol L^–1^ (3 equiv).

The linear section of the sigmoidal curve is crucial for quantitative measurements and for calibration [36]. The intersections of the slope at the point of inflection with the asymptote define the dynamic concentration range for which the assay delivers reliable quantitative data (Figure 4, solid line). The use of 3-fold stoichiometric excess of Eu^III^ in relation to **Bn** results in a variation in the absorption at 536 nm of 0.2 and a dynamic concentration range between 2 and 25 µmol L^–1^ of CaDPA. As a proof-of-concept, this condition was defined as default for the quantification of CaDPA because the minimum amount of lanthanide necessary to provide reliable results is used. In this condition, the following calibration curve: [CaDPA] (mol L^–1^) = (9.4±0.5)×10^–5^ ΔAbs^536 nm^ (Adj-R^2^ = 0.983, *N* = 5, Figure S4) was constructed for the determination of the concentration of CaDPA from the variation in the absorbance at 536 nm, if appropriate sample dilution is chosen. The LOD in this experimental condition is (2.2±1.1)×10^–6^ mol L^–1^, which is lower than the reported infectious dosage of spores (6×10^–5^ mol L^–1^ of CaDPA) [9], [37] and comparable to the LOD of more complex methods, such as surface-enhanced RAMAN spectroscopy on silver-coated silicon nanowire arrays (4×10^–6^ mol L^–1^) [37] and ratiometric fluorescent detection (0.2×10^–6^ mol L^–1^) [38].

Finally, the selectivity of the method was tested by varying the concentration of the following ligands: CaDPA, acetic acid, benzoic acid, phthalic acids, picolinic acids, nicotinic acids, isonicotinic acid and inorganic phosphate (Figure S5). No false positive was produced for any aromatic acid as well as for acetic acid, indicating the high selectivity of the method for DPA. Phosphate was included in the study because it has been reported to produce false positives and false negatives in assays based on lanthanide complexes, impairing the analysis of complex samples [13], [14], [39], [40]. As most lanthanide complexes, [Eu(Bn)]^+^ is sensitive to the presence of phosphate [39].

### Quantification of Bacterial Spores

**Figure 5:**
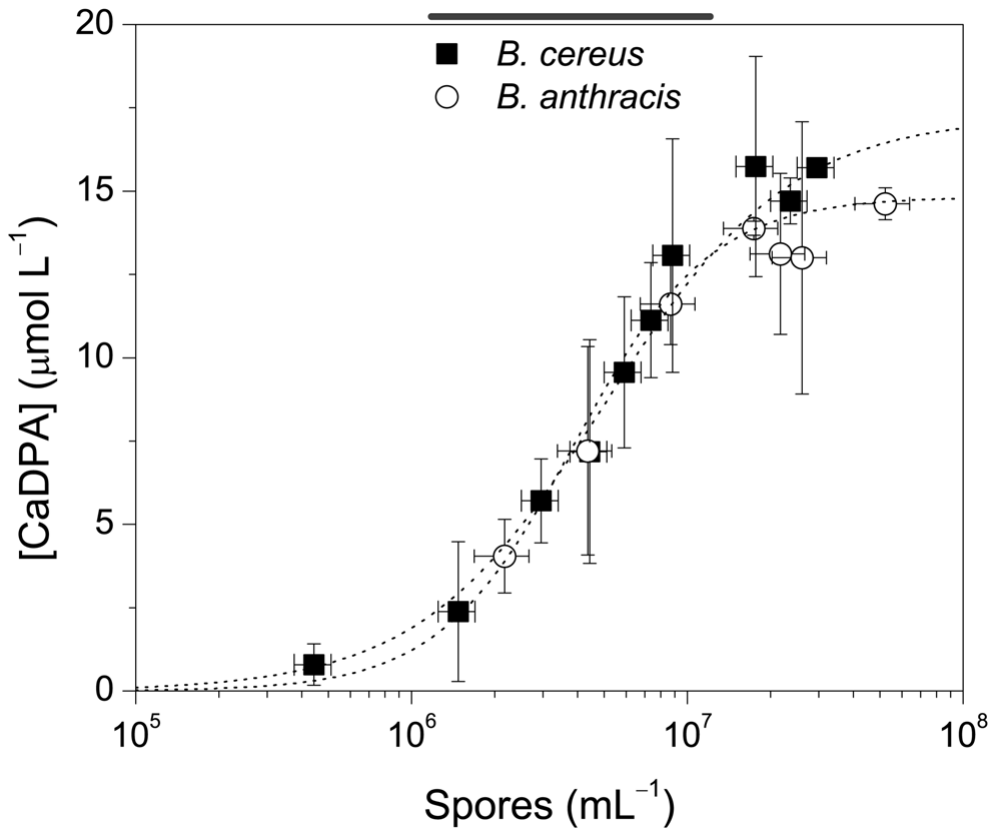
Quantification of bacteria of the genus *Bacillus* by the amount of CaDPA released upon thermal treatment. Vertical and horizontal error bars indicate uncertainties in spore counting and CaDPA quantification (sd, *N* = 3). The concentration of spores is in the log scale for clarity. [**Bn**] = 5.8 µmol L^–1^, [EuCl_3_] = 17.4 µmol L^–1^ (3 equiv) in MOPS buffer pH = 7.5.

Spores of *B. anthracis* and *B. cereus* were quantified using the plate count method (*see* Methods) and subjected to thermal treatment (autoclave) to induce the release of CaDPA. The concentration of CaDPA was determined using the colorimetric method and correlated to the concentration of spores (spores mL^–1^) (Figure 5). The uncertainty observed in the final result might be partially due to the plate count method because one single colony is not necessarily generated from a single spore [41]. However, the contribution of this to the overall uncertainty would be small. Other sources might come from sample preparation, such as extraction efficiency of CaDPA from spores.

Control experiments indicate that CaDPA can only be detected in spore suspensions subjected to thermal treatment (Figure S6). Furthermore, after monitoring the germination, we assume that most CaDPA is released from the spores upon thermal treatment (data not shown). The LOD determined for both *B. cereus* and *B. anthracis* is (1.1±0.3)×10^6^ spores mL^–1^. From the average slope of the linear section of the sigmoidal curves (Figure S7), we estimate that each spore has (1.6±0.5)×10^–15^ moles of CaDPA, i.e., (9.6±3.0)×10^8^ molecules of CaDPA per spore. Although this result is in reasonable agreement with the values reported using some other methods, including SERS and fluorescence detection, e.g., *Clostridium sporogenes* (3.7×10^8^ DPA molecules spore^–1^) [42] and *Bacillus* spp. spores (1.7×10^8^ to 6.3×10^8^ DPA molecules spore^–1^) [43], [44], [45], alternative methods requiring more sophisticate apparatus and data analysis may detect lower amounts of CaDPA in bacterial spores [13], [15], [46]. However, compared to the direct spectrophotometric detection of CaDPA, our method requires less sample preparation and is not susceptible to interferents absorbing in the middle UV region [47], [48].

The main limitation of the current methodology lies on the fact that the sensitivity depends on the molar absorptivity of **Bn**, implying that the detection limit of the present method cannot be easily enhanced. However, this approach introduces both the renewable natural pigment betanin as a biocompatible ligand for Eu^III^ and the [Eu(Bn)]^+^ complex as a green, low cost and fast compound for the detection of CaDPA and bacterial spores, as well as opens the perspective of exploring other betalains as a platform for the development of sensors.

## Conclusion

In this proof-of-concept study, we have shown that the orange complex formed between betanin, the main beetroot pigment, and Eu^III^ is sensitive to the presence of CaDPA, but not to other structurally similar pyridinic, aromatic, and acid ligands. The [Eu(Bn)]^+^ complex can be applied for the qualitative (on/off) and quantitative detection of CaDPA with a LOD of (2.2±1.1)×10^–6^ mol L^–1^. A colorimetric assay using 3 equiv of Eu^III^ in MOPS buffer was used to detect representative *Bacillus* species (*B. anthracis* or *B.cereus* spores) submitted to thermal treatment (autoclave). This low cost and ease of use approach indicates the potential use of betalains as sensors for biological applications.

## Supporting Information

Figure S1 **Absorption spectra of [Eu(Bn)]^+^ in MOPS buffer pH = 7.5 acquired over 5 d and decomposition kinetics monitored at 480 nm,** ***N*** **= 1.**
(TIF)

Figure S2 **Absorption of solutions of Bn and Eu^III^ (536 nm) at a fixed [Bn] = 5.75×10^–6^ mol L^–1^.**
(TIF)

Figure S3 **Spectroscopic data on the [Eu(Bn)]^+^ complex. (**A) Experimental Raman spectra (I_r_), second derivative of Raman Intensities relative to wavenumber (d^2^I_r_/dR_S_^2^) and theoretical intensities determined the B3LYP/6-31+G(d)/SDM level and corrected by a factor of 0.98 (I_r_^DFT^) and optimized structure of **Bn**; (B) Experimental Raman spectra (I_r_), second derivative of Raman Intensities relative to wavenumber (d^2^I_r_/dR_S_^2^) of [Eu(Bn)]^+^ and non-optimized illustration of a possible structure. [**Bn**] = 1×10^–4^ mol L^–1^, [EuCl_3_] = 3.6×10^–3^ mol L^–1^.
(TIF)

Figure S4 **Calibration curve for the determination of the [CaDPA] from the variation in absorbance at 536 nm.** The y-axis is in the log scale to show the sigmoidal profile of the curve and the linear fitting of the data. Curved lines are the confidence bands at the 95% level. Error bars represent the *sd* of triplicates. [**Bn**] = 5.8 µmol L^–1^, [EuCl_3_] = 17.4 µmol L^–1^ (3 equiv). [CaDPA] = (9.4±0.5)**×**10^–5^ ΔAbs^536 nm^ (Adj-R^2^ = 0.983, *N* = 5).
(TIF)

Figure S5 **Effect of the addition of carboxylic acids and phosphate to the [Eu(Bn)]^+^ complex monitored by the change in the absorption maxima of Bn.** [**Bn**] = 5.8 µmol L^–1^, [EuCl_3_] = 17.4 µmol L^–1^ (3 equiv), [analyte] = 69.6 µmol L^–1^ (12 equiv) in MOPS buffer pH = 7.5.
(TIF)

Figure S6 **Control experiments for the determination of CaDPA in samples containing spores of** ***B. cereus*** **not submitted to thermal treatment.** [**Bn**] = 5.8 µmol L^–1^, [EuCl_3_] = 17.4 µmol L^–1^ (3 equiv) in MOPS buffer pH = 7.5, *N* = 1.
(TIF)

Figure S7 **Quantification of bacteria of the genus** ***Bacillus*** **by the amount of CaDPA released upon thermal treatment for the determination of the amount of CaDPA per spore.** The red dotted line is the linear data fitting and the green lines are the confidence bands at 95% confidence level. Vertical and horizontal error bars indicate uncertainties in spore counting and CaDPA quantification (sd, *N* = 3). [**Bn**] = 5.8 µmol L^–1^, [EuCl_3_] = 17.4 µmol L^–1^ (3 equiv) in MOPS buffer pH = 7.5.
(TIF)

Table S1 **Stability constants determined using Eqs. (1) and (2) and the corresponding concentration of Eu^III^.**
(DOCX)

File S1 **Supplementary methods.**
(DOCX)
