# Supplementary figures and images for: Beetroot-Pigment-Derived Colorimetric Sensor for Detection of Calcium Dipicolinate in Bacterial Spores
Source: PLoS One. 2013 Sep 3;8(9):e73701. doi: 10.1371/journal.pone.0073701 (PMC3760816; doi:10.1371/journal.pone.0073701)

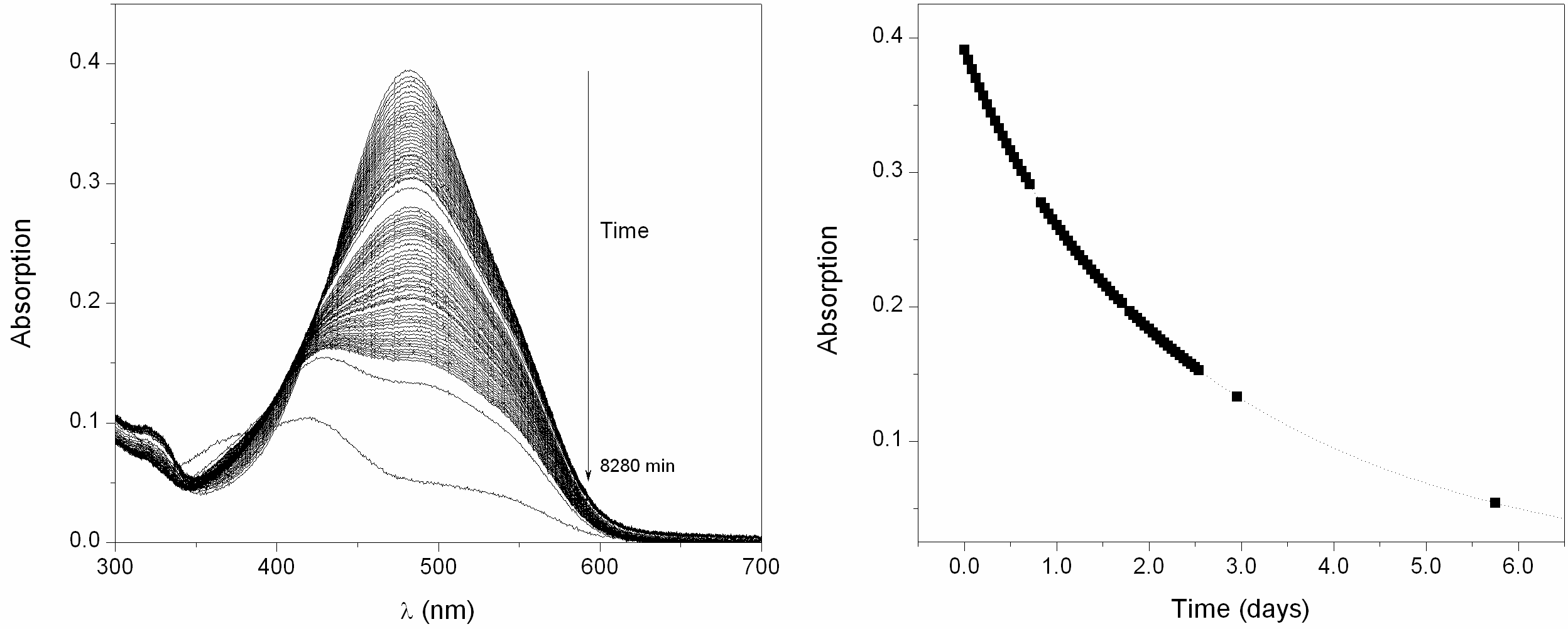

Supplement: Figure S1 — Absorption spectra of [Eu(Bn)]+ in MOPS buffer pH = 7.5 acquired over 5 d and decomposition kinetics monitored at 480 nm, N = 1. (TIF) [file pone.0073701.s001.tif]

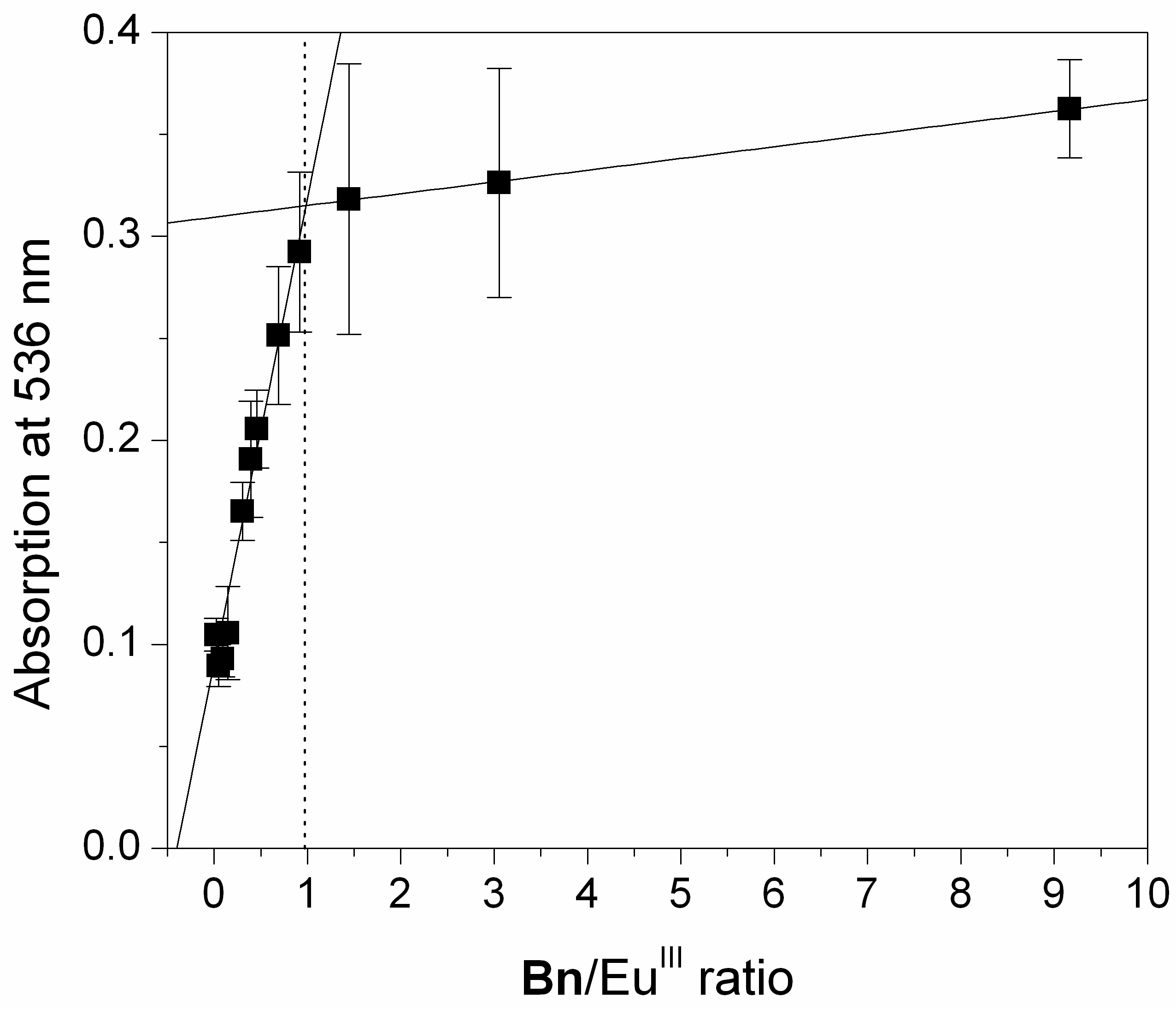

Supplement: Figure S2 — Absorption of solutions of Bn and EuIII (536 nm) at a fixed [Bn] = 5.75×10^–6^ mol L–1. (TIF) [file pone.0073701.s002.tif]

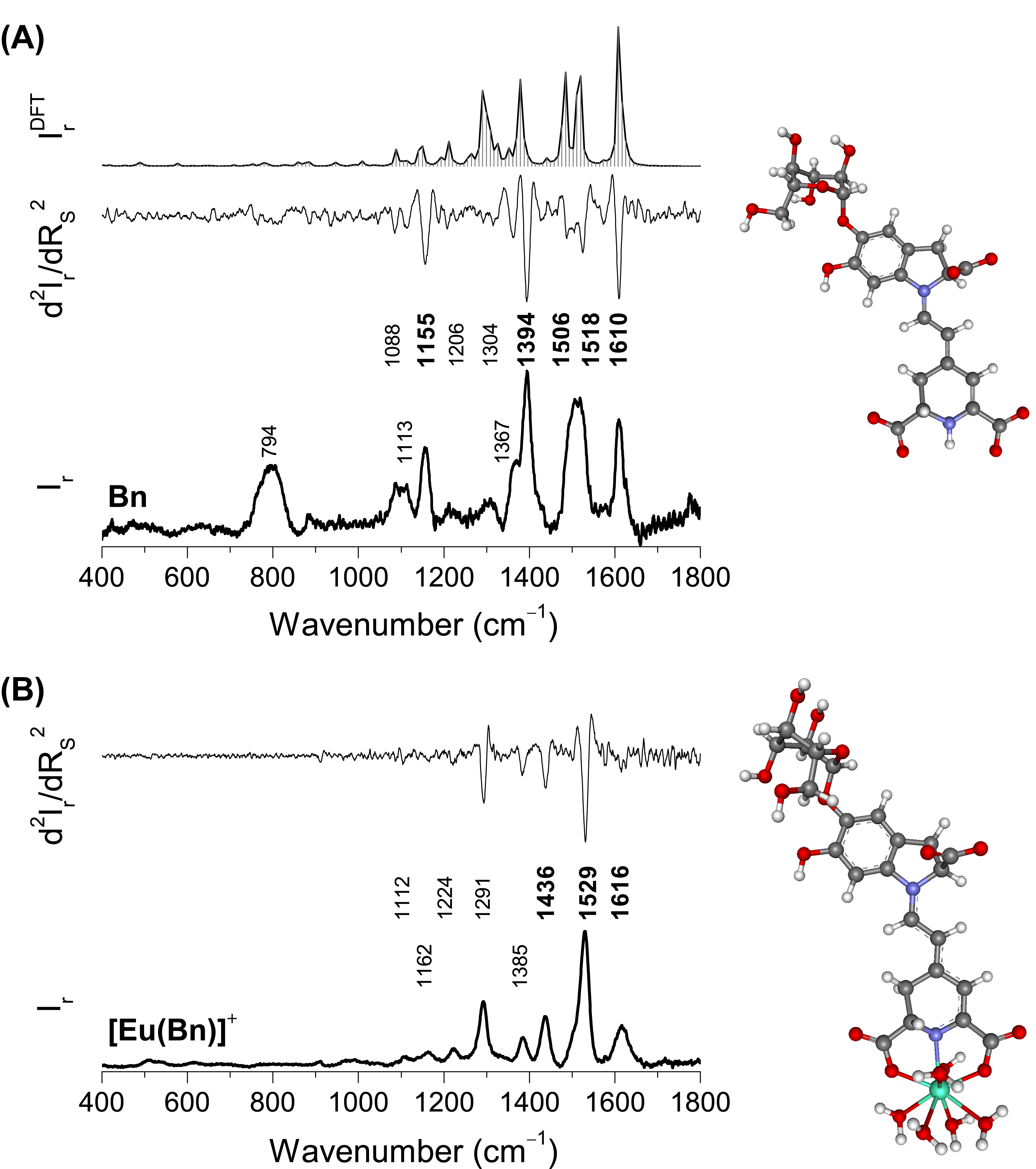

Supplement: Figure S3 — Spectroscopic data on the [Eu(Bn)]+ complex. (A) Experimental Raman spectra (Ir), second derivative of Raman Intensities relative to wavenumber (d2Ir/dRS 2) and theoretical intensities determined the B3LYP/6-31+G(d)/SDM level and corrected by a factor of 0.98 (Ir DFT) and optimized structure of Bn; (B) Experimental Raman spectra (Ir), second derivative of Raman Intensities relative to wavenumber (d2Ir/dRS 2) of [Eu(Bn)]+ and non-optimized illustration of a possible structure. [Bn] = 1×10^–4^ mol L–1, [EuCl3] = 3.6×10^–3^ mol L–1. (TIF) [file pone.0073701.s003.tif]

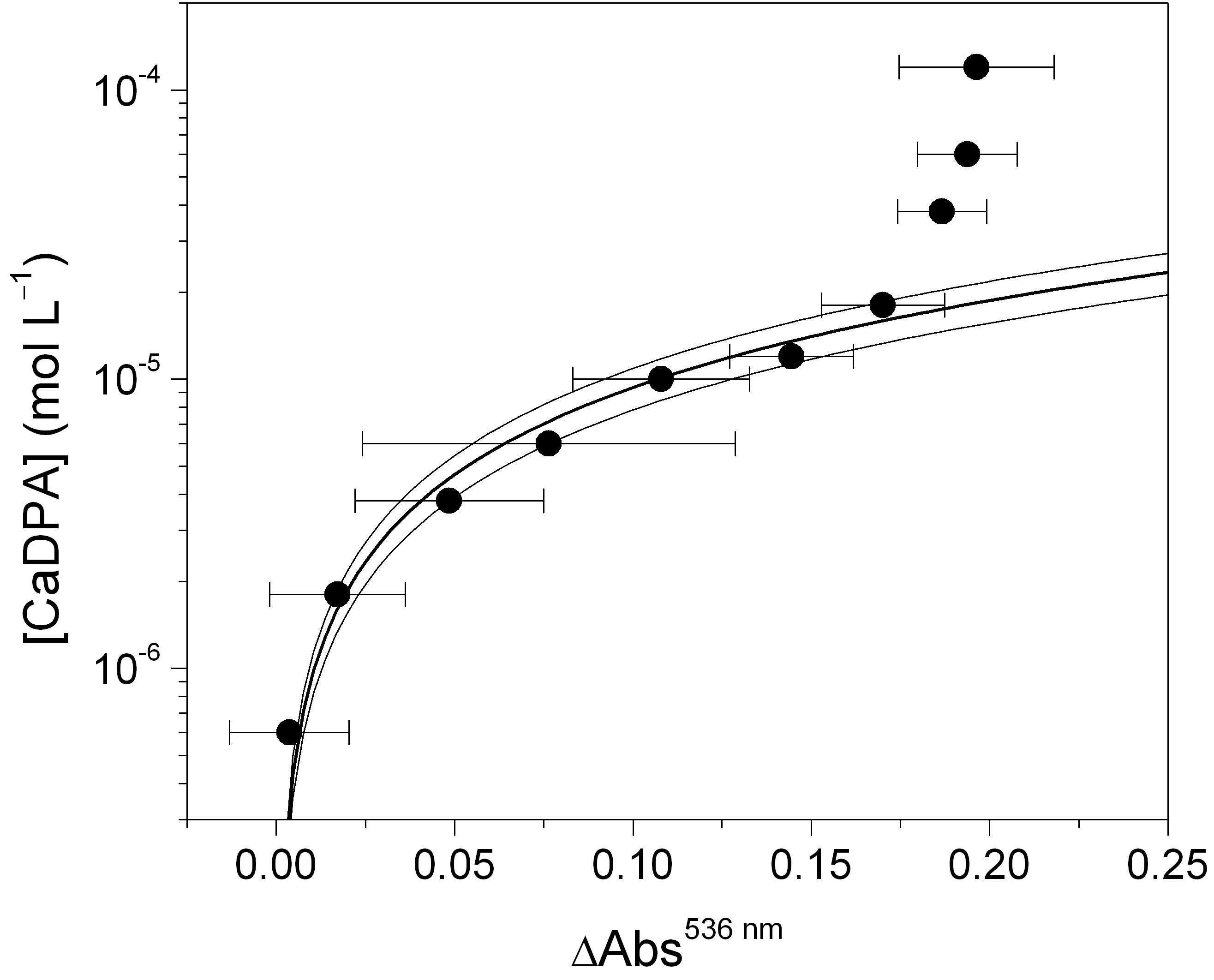

Supplement: Figure S4 — Calibration curve for the determination of the [CaDPA] from the variation in absorbance at 536 nm. The y-axis is in the log scale to show the sigmoidal profile of the curve and the linear fitting of the data. Curved lines are the confidence bands at the 95% level. Error bars represent the sd of triplicates. [Bn] = 5.8 µmol L–1, [EuCl3] = 17.4 µmol L–1 (3 equiv). [CaDPA] = (9.4±0.5)×10–5 ΔAbs536 nm (Adj-R2 = 0.983, N = 5). (TIF) [file pone.0073701.s004.tif]

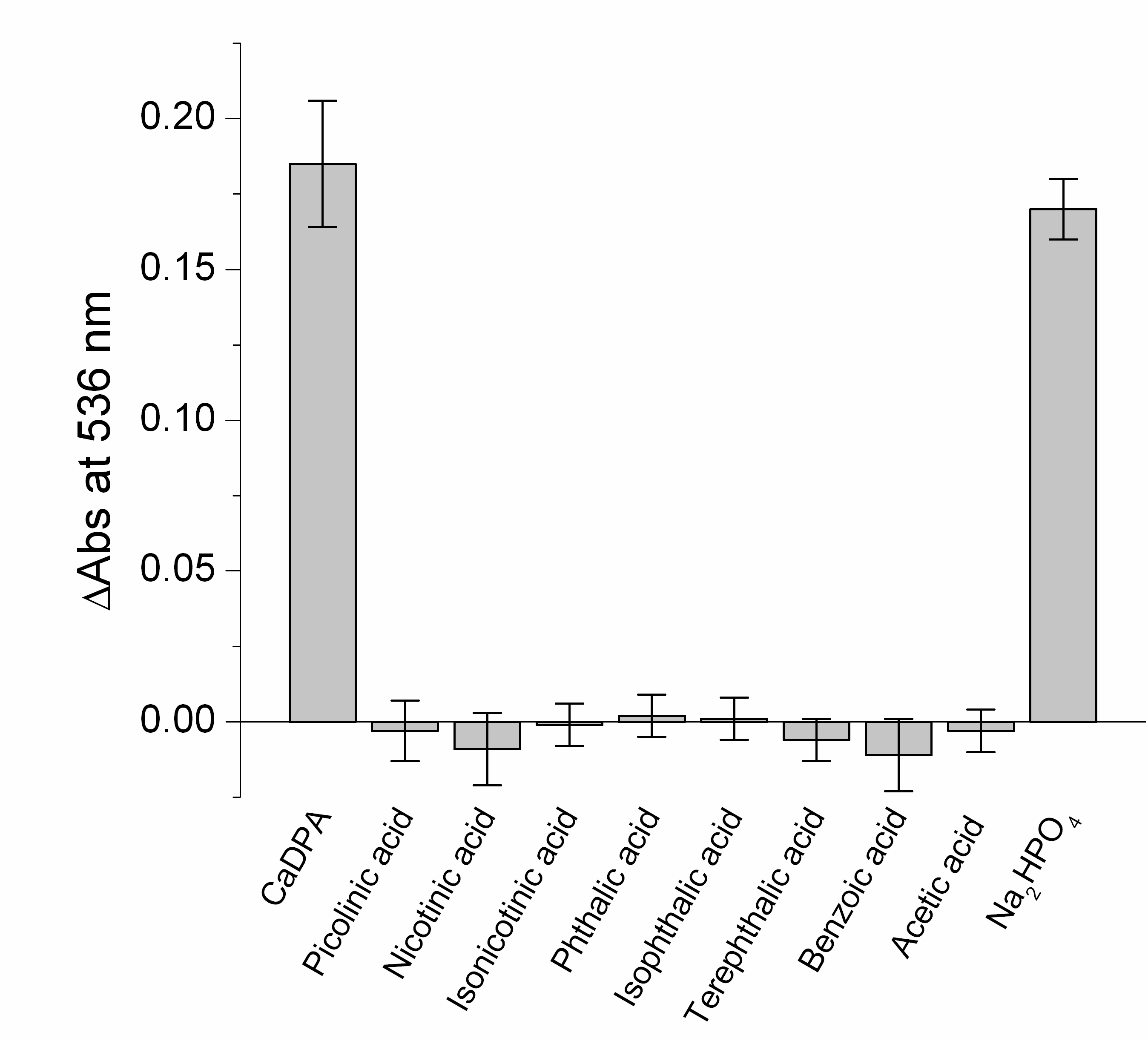

Supplement: Figure S5 — Effect of the addition of carboxylic acids and phosphate to the [Eu(Bn)]+ complex monitored by the change in the absorption maxima of Bn. [Bn] = 5.8 µmol L–1, [EuCl3] = 17.4 µmol L–1 (3 equiv), [analyte] = 69.6 µmol L–1 (12 equiv) in MOPS buffer pH = 7.5. (TIF) [file pone.0073701.s005.tif]

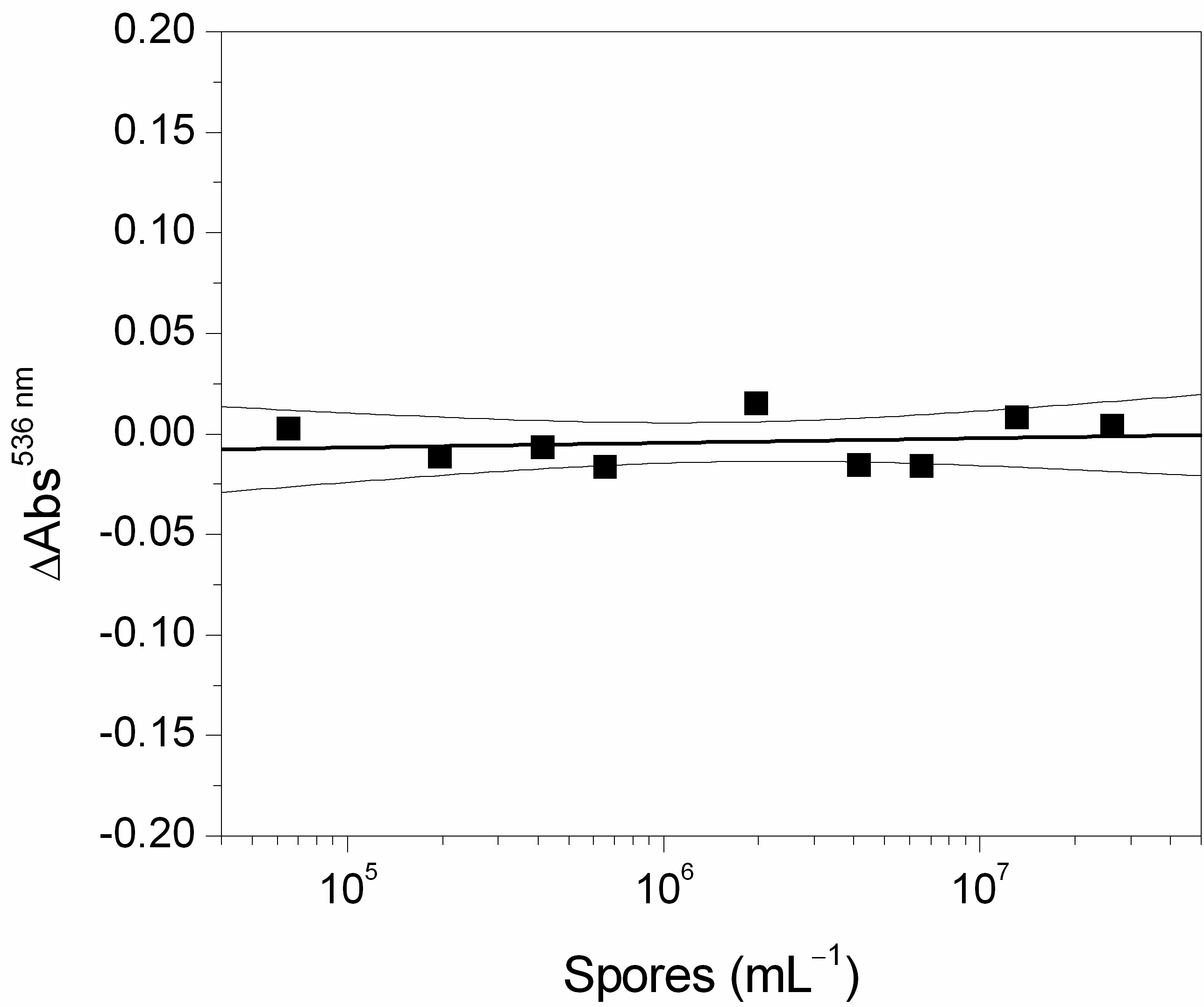

Supplement: Figure S6 — Control experiments for the determination of CaDPA in samples containing spores of B. cereus not submitted to thermal treatment. [Bn] = 5.8 µmol L–1, [EuCl3] = 17.4 µmol L–1 (3 equiv) in MOPS buffer pH = 7.5, N = 1. (TIF) [file pone.0073701.s006.tif]
