# Supplementary material for: Beetroot-Pigment-Derived Colorimetric Sensor for Detection of Calcium Dipicolinate in Bacterial Spores
Source: PLoS One. 2013 Sep 3;8(9):e73701. doi: 10.1371/journal.pone.0073701 (PMC3760816; doi:10.1371/journal.pone.0073701)
Supplement: Table S1 — Stability constants determined using Eqs. (1) and (2) and the corresponding concentration of EuIII. (DOCX) [file pone.0073701.s008.docx]

**Table S1.** Stability constants determined using Eqs. (1) and (2) and the corresponding concentration of Eu^III^.

| **[Eu^III^] (mol L^–1^)** | **K (L mol^–1^)** |
| --- | --- |
| 1.82 × 10^–6^ | 1.26 × 10^5^ |
| 3.85 × 10^–6^ | 5.98 × 10^4^ |
| 6.08 × 10^–6^ | 6.72 × 10^4^ |
| 8.11 × 10^–6^ | 1.04 × 10^5^ |
| 1.22 × 10^–5^ | 1.29 × 10^5^ |
| 1.42 × 10^–5^ | 1.33 × 10^5^ |
| 1.82 × 10^–5^ | 1.47 × 10^5^ |
| 3.85 × 10^–5^ | 2.31 × 10^5^ |
| 6.08 × 10^–5^ | 2.38 × 10^5^ |
| 1.22 × 10^–4^ | 1.38 × 10^5^ |
| Mean ± sd | (1.4 ± 0.6) × 10^5^ |

Parameters: [L]_0_ = 5.75 × 10^–6^ mol L^–1^; ε**_Bn_**^536 nm^ = 65,000 mol L^–1^ cm^–1^; ε_[Eu(_**_Bn_**_)]+_^536 nm^ = 13,000 mol L^–1^ cm^–1^.
