# Supplementary material for: Beetroot-Pigment-Derived Colorimetric Sensor for Detection of Calcium Dipicolinate in Bacterial Spores
Source: PLoS One. 2013 Sep 3;8(9):e73701. doi: 10.1371/journal.pone.0073701 (PMC3760816; doi:10.1371/journal.pone.0073701)
Supplement: File S1 — Supplementary methods. (DOCX) [file pone.0073701.s009.docx]

**SUPPORTING INFORMATION**

**Beetroot-pigment-derived colorimetric sensor for detection of calcium dipicolinate in bacterial spores**

Letícia Christina Pires Gonçalves, Sandra Da Silva, Paul C. DeRose, Rômulo Augusto Ando and Erick Leite Bastos*

# SUPPLEMENTARY METHODS

## 1.1 Chemicals

2,6-Pyridinedicarboxylic acid (dipicolinic acid, DPA), europium chloride (EuCl_3_), 3-(*N*-morpholino)propanesulfonic acid (MOPS), potassium phosphates (K_3_PO_4_, K_2_HPO_4_ and KH_2_PO_4_), magnesium sulfate heptahydrate (MgSO_4_·7H_2_O), manganese sulfate (MnSO_4_), manganese chloride tetrahydrate (MnCl_2_ ·4H_2_O), zinc sulfate (ZnSO_4_), iron (II) sulfate heptahydrate (FeSO_4_·7H_2_O), calcium chloride dihydrate (CaCl_2_ ·2H_2_O), sodium hydroxide (NaOH), potassium chloride (KCl), trifluoroacetic acid (TFA), acetic acid (HOAc), silicagel 90 C_18_-RP (230-400 mesh), benzoic acid, phthalic acid, isophthalic acid, terephthalic acid, picolinic acid, nicotinic acid and isonicotinic acid were obtained from Sigma-Aldrich. Methanol (MeOH) and acetonitrile (MeCN) were HPLC-grade and were obtained from Merck. Bacto™ peptone was obtained from Difco (VGDINC, USA). All solutions were prepared using deionized water (water, 18.2 MΩ∙cm at 25 ºC, Milli-Q, Millipore). LB agar, glucose and Tween 80 were obtained from Fisher scientific and phosphate buffered saline (PBS) from Invitrogen.

## 1.2 Purification of betanin

Extraction, purification and characterization of betanin have been carried out as described previously [[1](#_ENREF_1)]. Briefly, beetroots (*Beta vulgaris* subsp. *vulgaris* var. *vulgaris,* 0.5 kg) were peeled, sliced and homogenized in a centrifugal juice extractor (Phillips–Walita, RI1858) at maximum speed. The juice was centrifuged (3500 rpm, 30 min, 25 ºC), filtered (Whatman qualitative filter paper, grade 4) and the supernatant was stored at –20 ºC and used within 5 d. Betanin/isobetanin mixture was purified from beetroot juice by reversed-phase column chromatography (silica gel 90 C18 (20 g) conditioned and eluted with water at flow rate of 0.3 mL min^–1^). Betanin stock solution were prepared in water and the concentration was determined by assuming a molar absorption coefficient (*ε*) of 6.5 × 10^4^ L mol^–1^ cm^–1^ at 536 nm [[2](#_ENREF_2)] after analytical RP-HPLC and HPLC-DAD-ESI(+)-MS/MS analysis.

## 1.3 Solution of calcium dipicolinate

The stock solution of CaDPA (1.0 × 10^–3^ mol L^–1^) was prepared by dissolving DPA (8.3 mg, 50 μmol) and CaCl_2_ (5.5 mg, 50 μmol) in 50 mL of MOPS buffer pH = 7.5 at room temperature [[3](#_ENREF_3)].

## 1.4 Buffers and culture media

### 1.4.1 Peptone glucose sporulation medium (PGSM)

PGSM solid media was prepared by dissolving Bacto™ peptone (7.5 g), glucose (1.0 g), KH_2_PO_4_ (3.4 g), K_2_HPO_4_ (4.35 g) and agar (15 g) into 1.0 L of water followed by autoclaving. Post-autoclaving, 1.0 mL of a solution containing MgSO_4_ (2.46 g), MnSO_4_ (0.04 g), ZnSO_4_ (0.28 g) and FeSO_4_ (0.40 g) per 100 mL water and 1.0 mL of a solution of CaCl_2_ (3.66 g CaCl_2_ per 100 mL water) were added to the media.

### 1.4.2 Modified Schaeffer media

Sporulation media was prepared by dissolving nutrient broth (8 g, Difco Bacto peptone), MgSO_4_·7H_2_O (0.51 g), MnCl_2_·4H_2_O (3 × 10^–3^ g), KCl (0.97 g), FeSO_4_·7H_2_O (0.55 × 10^–3^ g), CaCl_2_·2H_2_O (0.2 g) and 1.5% agar in 1 L sterile water and adjusting the pH to 6.9.

### 1.4.3 PBST (Phosphate buffered saline + Tween 80)

PBST solution (pH = 7.4, 10 mmol L^–1^, 0.4% v/v Tween 80) was prepared by dissolving 4 mL Tween 80 in 1 L of phosphate buffered saline (0.1 mol L^–1^, pH = 7.4). The mixture was stirred at room temperature until Tween 80 was completely dissolved and the resulting solution was stored at room temperature.

### 1.4.4 MOPS

MOPS buffer solution (pH = 7.5, 10 mmol L^–1^) was prepared by dissolving 1.04 g of 3-(*N*-morpholino)propanesulfonic acid (p*K*_a_ = 7.2) in 475 mL of water. The pH was adjusted to 7.5 with a solution of NaOH (1 mol L^–1^) and the volume was completed to 500 mL.

## 1.5 Spectrophotometric measurements

### 1.5.1 UV-Vis spectroscopy

Absorption spectra were recorded in the UV–Vis region of the electromagnetic spectra (250 – 700 nm) at 25 ± 1 ºC on a Varian Cary 50 Bio spectrophotometer equipped with a Peltier thermostatted cell holder. Alternatively, absorption intensities at 536 nm were recorded at 25 ± 1 ºC on a SpectraMax M2 & M2e multi-mode microplate reader (Molecular Devices) using sterile transparent 96-well microplates (final volume = 200 μL).

### 1.5.2 RAMAN spectroscopy

The resonance Raman spectra were obtained in a triple spectrometer Jobin-Yvon T64000 equipped with a charge-coupled device (CCD Symphony Horiba Jobin-Yvon) detector at 90º scattering configuration in a typical resolution of 2 cm^–1^ (grating of 1800 lines cm^–1^ and 200 µm of slit). The excitation wavelengths employed were 514.5 and 476.5 nm from a mixed Ar^+^/Kr^+^ ion laser (Coherent Innova 70C) at laser power of 20 mW on the samples placed in a NMR tube coupled to a rotator shaft to avoid local heating.

## 1.6 Computational details

The ground state geometry of **Bn** was fully optimized employing the density functional theory (DFT) at the B3LYP/6-31+g(d)/SMD level [[4](#_ENREF_4),[5](#_ENREF_5),[6](#_ENREF_6),[7](#_ENREF_7)]. Vibrational analyses revealed no imaginary frequencies, indicating that the optimized geometries were in a minimum of the potential energy surface. The geometry optimizations and the vibrational spectra were performed with the aid of the Gaussian 09 software [[8](#_ENREF_8)]. The theoretical Raman spectra were plotted using 5 cm^−1^ of bandwidth and a 0.98 scaling factor was employed on the calculated harmonic vibrational wavenumbers to compare the results with the experimental data.

## 1.7 Determination of stability constants [[9](#_ENREF_9)]

For a simple metal–ligand complexation:

where, L: ligand; M: metal; C: complex; a and b are the stoichiometric factors; [L]_0_ and [M]_0_: initial total concentration of the ligand and the metal, respectively; [L], [M] and [C]: equilibrium concentration of the ligand, the metal and the complex, respectively.

Substituting Eqs. III and IV in Eq. II:

For the determination of *K* by UV/Vis spectrometry, it is necessary to determine the [C]. Consider:

where, $A_{obs}^{\lambda}$ is the observed absorbance at a given wavelength and $A_{L}^{\lambda},A_{M}^{\lambda} \mathrm{and} A_{C}^{\lambda}$ , and $\varepsilon_{L}^{\lambda},\varepsilon_{M}^{\lambda} \mathrm{and} \varepsilon_{C}^{\lambda}$are the absorbances and molar absorption coefficient of the ligand, metal and complex at the same wavelength, respectively.

Eq. VI is combined to Eqs. VII, VIII and IX to yield:

rearranging:

In case the metal does not absorbs at the wavelength λ, Eq. XI is reduced to:

**Coordinates: Bn**

| C | 0.028756000000 | 0.616365000000 | -0.104542000000 |
| --- | --- | --- | --- |
| N | –1.296782000000 | 1.074557000000 | 0.088667000000 |
| C | 0.934122000000 | 1.582125000000 | 0.326668000000 |
| C | 0.449711000000 | –0.608833000000 | –0.626314000000 |
| C | –1.283542000000 | 2.503000000000 | 0.489835000000 |
| C | –2.403233000000 | 0.355953000000 | –0.118994000000 |
| C | 0.199361000000 | 2.779071000000 | 0.877034000000 |
| C | 2.304964000000 | 1.338174000000 | 0.250726000000 |
| C | 1.819646000000 | –0.846658000000 | –0.706851000000 |
| C | –1.748763000000 | 3.454859000000 | –0.647418000000 |
| C | –3.706506000000 | 0.802220000000 | 0.071081000000 |
| C | 2.756085000000 | 0.121930000000 | –0.274404000000 |
| O | 2.331945000000 | –2.020958000000 | –1.214479000000 |
| O | –1.743778000000 | 3.035108000000 | –1.837554000000 |
| C | –4.845148000000 | 0.011565000000 | –0.167684000000 |
| O | 4.073294000000 | –0.248749000000 | –0.406758000000 |
| C | –4.772474000000 | –1.374145000000 | –0.778689000000 |
| C | –6.130447000000 | 0.536616000000 | 0.051321000000 |
| C | 5.122722000000 | 0.719129000000 | –0.236419000000 |
| C | –5.957053000000 | –2.263340000000 | –0.371905000000 |
| C | –7.273471000000 | –0.226512000000 | –0.190811000000 |
| C | 5.618486000000 | 0.754765000000 | 1.222167000000 |
| O | 6.142260000000 | 0.422915000000 | –1.158346000000 |
| N | –7.200342000000 | –1.519429000000 | –0.515578000000 |
| C | –5.789397000000 | –2.877057000000 | 1.056417000000 |
| C | –8.690010000000 | 0.355516000000 | –0.096788000000 |
| C | 6.475331000000 | –0.465243000000 | 1.595608000000 |
| O | 6.358987000000 | 1.962675000000 | 1.431740000000 |
| C | 6.997866000000 | –0.720132000000 | –0.914488000000 |
| O | –4.698566000000 | –3.491798000000 | 1.248441000000 |
| O | –6.734474000000 | –2.763742000000 | 1.886343000000 |
| O | –8.794074000000 | 1.588607000000 | 0.148666000000 |
| O | –9.635749000000 | –0.465724000000 | –0.276853000000 |
| C | 7.545575000000 | –0.736868000000 | 0.520246000000 |
| O | 5.608100000000 | –1.584269000000 | 1.786757000000 |
| C | 6.332222000000 | –2.026164000000 | –1.369022000000 |
| O | 8.521802000000 | 0.309524000000 | 0.706971000000 |
| O | 7.385523000000 | –2.989301000000 | –1.550545000000 |
| H | –0.243006000000 | –1.368996000000 | –0.975111000000 |
| H | –1.949273000000 | 2.636004000000 | 1.344188000000 |
| H | –2.221315000000 | –0.660998000000 | –0.446524000000 |
| H | 0.307397000000 | 2.845963000000 | 1.965885000000 |
| H | 0.553609000000 | 3.724143000000 | 0.454942000000 |
| H | 3.002138000000 | 2.086028000000 | 0.611890000000 |
| H | –3.879072000000 | 1.815117000000 | 0.424726000000 |
| H | 1.599937000000 | –2.601112000000 | –1.488661000000 |
| H | –4.771858000000 | –1.252353000000 | –1.871140000000 |
| H | –3.853609000000 | –1.900267000000 | –0.518598000000 |
| H | –6.254107000000 | 1.564220000000 | 0.372322000000 |
| H | 4.758797000000 | 1.705990000000 | –0.528984000000 |
| H | –5.997815000000 | –3.115797000000 | –1.056205000000 |
| H | 4.752244000000 | 0.802672000000 | 1.886297000000 |
| H | –8.082196000000 | –2.023654000000 | –0.538570000000 |
| H | 6.993347000000 | –0.232883000000 | 2.533828000000 |
| H | 7.258985000000 | 1.823957000000 | 1.072977000000 |
| H | 7.842890000000 | –0.549172000000 | –1.590275000000 |
| H | 8.010526000000 | –1.711637000000 | 0.708881000000 |
| H | 6.139760000000 | –2.325516000000 | 2.124591000000 |
| H | 5.607384000000 | –2.395566000000 | –0.640864000000 |
| H | 5.821039000000 | –1.840411000000 | –2.320977000000 |
| H | 9.207390000000 | 0.232186000000 | 0.020530000000 |
| H | 6.974554000000 | –3.854574000000 | –1.712964000000 |
| O | –2.066105000000 | 4.619803000000 | –0.262439000000 |

**References**

1. Gonçalves LCP, Trassi MAD, Lopes NB, Dörr FA, dos Santos MT, et al. (2012) A comparative study of the purification of betanin. Food Chem 131: 231-238.

2. Schwartz SJ, Von Elbe JH (1980) Quantitative determination of individual betacyanin pigments by high-performance liquid chromatography. J Agric Food Chem 28: 540-543.

3. Peng L, Chen D, Setlow P, Li Y-q (2009) Elastic and Inelastic Light Scattering from Single Bacterial Spores in an Optical Trap Allows the Monitoring of Spore Germination Dynamics. Anal Chem 81: 4035-4042.

4. Becke AD (1993) Density-functional thermochemistry. III. The role of exact exchange. J Chem Phys 98: 5648-5652.

5. Lee C, Yang W, Parr RG (1988) Development of the Colle-Salvetti correlation-energy formula into a functional of the electron density. Phys Rev B Condens Matter 37: 785-789.

6. Marenich AV, Cramer CJ, Truhlar DG (2009) Performance of SM6, SM8, and SMD on the SAMPL1 Test Set for the Prediction of Small-Molecule Solvation Free Energies. J Phys Chem B 113: 4538-4543.

7. Marenich AV, Cramer CJ, Truhlar DG (2009) Universal Solvation Model Based on Solute Electron Density and on a Continuum Model of the Solvent Defined by the Bulk Dielectric Constant and Atomic Surface Tensions. J Phys Chem B 113: 6378-6396.

8. Frisch MJ, Trucks GW, Schlegel HB, Scuseria GE, Robb MA, et al. (2009) Gaussian 09, Revision B.01. Wallingford CT.

9. Hirose K (2001) A Practical Guide for the Determination of Binding Constants. J Inclusion Phenom Macrocyclic Chem 39: 193-209.
